# Recombinant L-Asparaginase from *Zymomonas mobilis*: A Potential New Antileukemic Agent Produced in *Escherichia coli*

**DOI:** 10.1371/journal.pone.0156692

**Published:** 2016-06-02

**Authors:** Karen Einsfeldt, Isis Cavalcante Baptista, Juliana Christina Castanheira Vicente Pereira, Isabele Campos Costa-Amaral, Elaine Sobral da Costa, Maria Cecília Menks Ribeiro, Marcelo Gerardin Poirot Land, Tito Lívio Moitinho Alves, Ariane Leites Larentis, Rodrigo Volcan Almeida

**Affiliations:** 1 Programa de Engenharia Química, COPPE, Universidade Federal do Rio de Janeiro (UFRJ), Rio de Janeiro, Brazil; 2 Programa de Pós-Graduação em Bioquímica, Instituto de Química, Universidade Federal do Rio de Janeiro (UFRJ), Rio de Janeiro, Brazil; 3 Programa de Pós-Graduação em Clínica Médica, Universidade Federal do Rio de Janeiro (UFRJ), Rio de Janeiro, Brazil; 4 Programa de Saúde Pública e Meio Ambiente, Escola Nacional de Saúde Pública Sergio Arouca (ENSP), Centro de Estudos da Saúde do Trabalhador e Ecologia Humana (CESTEH), Fundação Oswaldo Cruz (FIOCRUZ), Rio de Janeiro, Rio de Janeiro, Brazil; Institute of Biochemistry and Biotechnology, TAIWAN

## Abstract

L-asparaginase is an enzyme used as a chemotherapeutic agent, mainly for treating acute lymphoblastic leukemia. In this study, the gene of L-asparaginase from *Zymomonas mobilis* was cloned in pET vectors, fused to a histidine tag, and had its codons optimized. The L-asparaginase was expressed extracellularly and intracellularly (cytoplasmically) in *Escherichia coli* in far larger quantities than obtained from the microorganism of origin, and sufficient for initial cytotoxicity tests on leukemic cells. The *in silico* analysis of the protein from *Z*. *mobilis* indicated the presence of a signal peptide in the sequence, as well as high identity to other sequences of L-asparaginases with antileukemic activity. The protein was expressed in a bioreactor with a complex culture medium, yielding 0.13 IU/mL extracellular L-asparaginase and 3.6 IU/mL intracellular L-asparaginase after 4 h of induction with IPTG. The cytotoxicity results suggest that recombinant L-asparaginase from *Z*. *mobilis* expressed extracellularly in *E*.*coli* has a cytotoxic and cytostatic effect on leukemic cells.

## Introduction

One of the main therapeutic enzymes of microbial origin, L-asparaginase (L-asparagine amino hydrolase, E.C. 3.5.1.1) is used as a chemotherapeutic agent to treat a number of lymphoproliferative disorders and lymphomas, especially acute lymphoblastic leukemia (ALL) [1,2]. L-asparaginase works by depleting the exogenous supply of L-asparagine to cells, since malignant cells synthesize L-asparagine more slowly than they need and depend on the exogenous supply of this amino acid. Meanwhile, normal cells are able to synthesize all the amino acid they need and are therefore not harmed by the use of L-asparaginase [3–6].

There are two types of bacterial L-asparaginase, type I and type II, but only type II has anti-leukemic activity. Type II L-asparaginases from bacteria are homotetramers composed of subunits with approximately 300 amino acids [7].

There are ALL treatments on the market comprising formulations of L-asparaginase obtained from *Escherichia coli* and *Erwinia chrysanthemi* and there is a formulation of polyethylene glycol-conjugated L-asparaginase [8,9]. The main problem of using L-asparaginase is clinical hypersensitivity developed by patients treated with unmodified forms of the enzyme. Studies on L-asparaginases from *E*. *coli* and *E*. *chrysanthemi* have found that both enzymes demonstrate immunogenicity [1], and have been used as alternatives for patients who have developed sensitivity to one or the other [6,10]. Another way of circumventing hypersensitivity is to use a different type of L-asparaginase (obtained from a different microorganism or a polyethylene glycol conjugate). This is why it is so important to find new sources of L-asparaginase with antileukemic activity.

The Chemical Engineering Program’s Bioprocesses Laboratory at COPPE, Federal University of Rio de Janeiro, Brazil, has used the bacterium *Zymomonas mobilis* for years to study the production of various substances, like ethanol, glyconic acid, sorbitol, lactobionic acid, and L-asparaginase [11–16]. The L-asparaginase obtained from *Z*. *mobilis* is a periplasmic enzyme whose K_m_ (Michaelis-Menten constant) has been estimated at 1.5x10^-5^ M [15], which demonstrates that L-asparaginase from *Z*. *mobilis* has a strong affinity to the substrate L-asparagine. This value is very close to the K_m_ of type II L-asparaginase from *E*. *coli*, which has antileukemic activity. L-asparaginase was obtained from *Z*. *mobilis* using liquid cultures in agitated flasks, yielding activity of 37.8 IU/g dry cell weight or 0.005 IU/mL medium in 33 h culture time [15]. The low yield of the enzyme from *Z*. *mobilis* has hampered the study and production of L-asparaginase from this microorganism. However, this obstacle could be overcome if the recombinant enzyme were produced in high concentrations using genetic engineering.

This study therefore aimed to clone and express L-asparaginase from *Z*. *mobilis* in *E*. *coli* using plasmids for cytoplasmic and extracellular expression, and to evaluate the cytotoxicity of this enzyme on leukemia cells. To our knowledge, this is the first time that L-asparaginase from *Z*. *mobilis* has been produced as a recombinant protein expressed in another host.

## Materials and Methods

### Chemicals and bacterial strains

Luria-Bertani broth (LB) was obtained from Sigma-Aldrich. Kanamycin was obtained from Gibco. IPTG (isopropyl β-D-1-thiogalactopyranoside) was obtained from Promega. Glucose was obtained from Vetec. *Escherichia coli* DH5α (Invitrogen Carlsbad, CA, USA) was used as the host for the cloning procedures. *E*. *coli* BL21 (DE3) (Invitrogen, Carlsbad, CA, USA) was used for expressing L-asparaginase.

### In silico characterization of the protein

The protein was characterized *in silico* using bio-computational tools. The molecular mass and size of the protein were estimated using the http://www.uniprot.org website, and the theoretical isoelectric point (pI) of the protein was estimated using the http://web.expasy.org/compute_pi/ website. SignalP 3.0 was used to ascertain the presence of possible signal sequences (signal peptides) in the enzyme’s amino acid sequence. The global alignment between the amino acid sequences of L-asparaginases types I and II was ascertained with the help of CLUSTAL W software (S1 Supplementary Material). The phylogenetic tree was constructed using Phylogeny.fr, a program available at http://phylogeny.lirmm.fr/phylo_cgi/simple_phylogeny.cgi [17].

### Synthesis of gene and cloning

**Fig 1:**
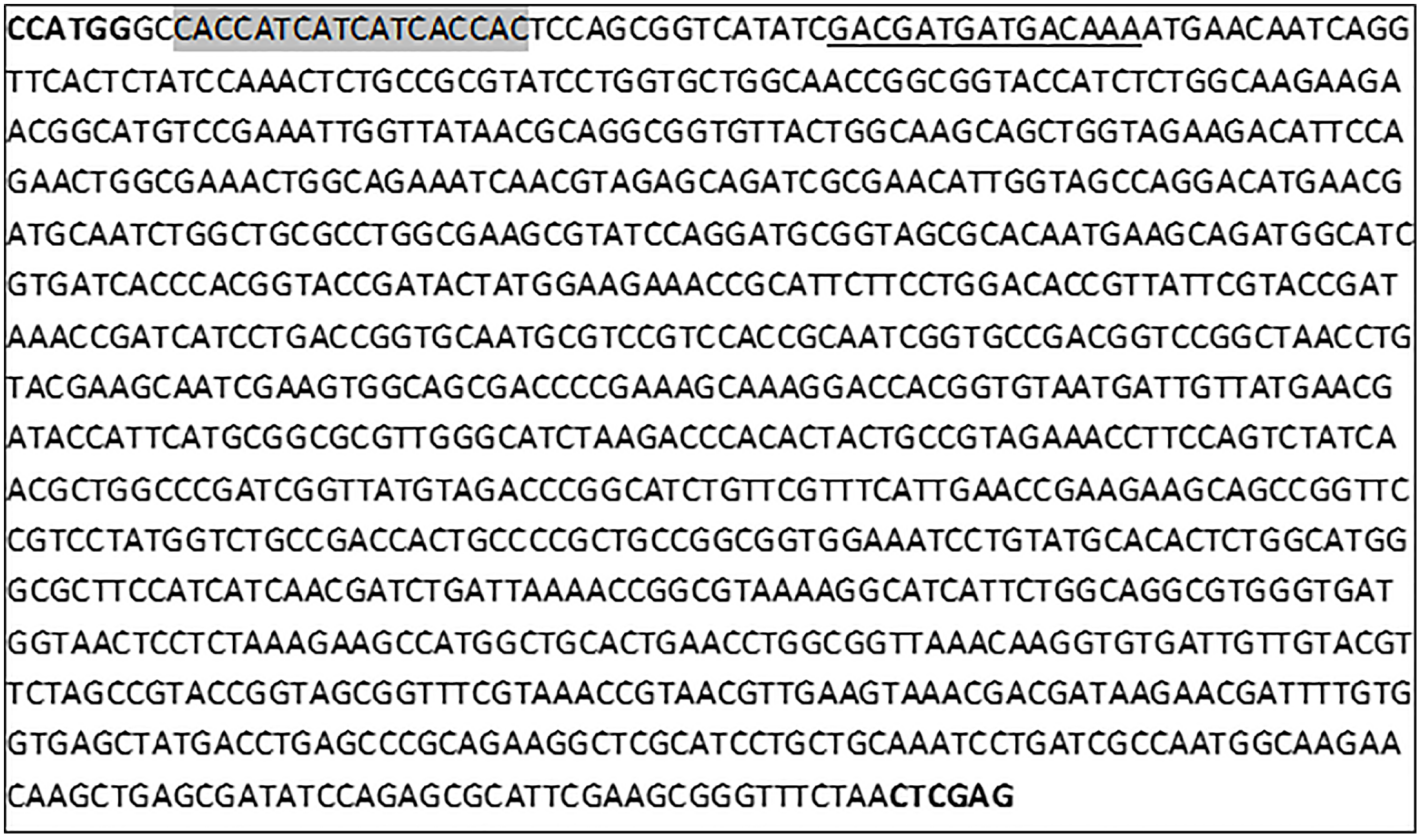
Sequence of the synthetic gene of L-asparaginase from *Z*. *mobilis* ATCC 31821. The nucleotides in bold are the sequences of enzymes *Nco*I and *Xho*I. The nucleotides marked in gray are the sequence that encodes the six histidines. The underscored nucleotides are the sequence that encodes the enterokinase cleavage site.

The gene that encodes L-asparaginase from *Z*. *mobilis* was chemically synthesized by Epoch Life Science Inc. It was designed from the sequence of 1101 nucleotides from the gene of *Z*. *mobilis* strain ATCC 31821 (Accession Code: NC_006526.2 of the GenBank database: http://www.ncbi.nlm.nih.gov/Genbank/index.html). The nucleotides that encode the first 29 amino acids of this sequence were removed, since they are part of a signal sequence (signal peptide). A sequence of nucleotides that encodes six histidines was added to the gene sequence that encodes the protein (without the signal peptide), enabling the expression of a fusion protein with the addition of a histidine tag at the N-terminus to facilitate its subsequent purification. An enterokinase cleavage site (Asp-Asp-Asp-Asp-Lys) was also added to remove the histidine tag of the recombinant protein produced. The codons used in the synthetic gene were optimized to the codons most frequently used in *E*. *coli*. The synthetic gene sequence can be seen in Fig 1. The synthetic gene, flanked by restriction enzymes *Nco*I and *Xho*I, was inserted into the vectors pET26b (Novagen) and pET28a (Novagen). The signal sequence (signal peptide) of pET26b is *pelB*. The vectors containing the synthetic gene were called pET26b/*ans* and pET28a/*ans*.

### L-asparaginase expression

Recombinant bacteria *E*. *coli* BL21 (DE3)/pET26b/ans and *E*. *coli* BL21 (DE3)/pET28a/ans were grown in Multifors 2 bioreactors (INFORS HT) with 400 mL LB medium supplemented with 1% glucose and 50 μg/mL kanamycin. The cultures were maintained at 37°C and agitated at a range of 200 to 800 rpm, maintaining a minimum pO_2_ of 60%, and a pH of 7.0 by the addition of 2 M NaOH and 1 M H_3_PO_4_. For the pre-inoculum, a 10 μL aliquot from a stab culture at -80°C of each of the recombinant bacteria was inoculated in 10 mL LB medium with 1% glucose and 50 μg/mL kanamycin. The pre-inoculum was incubated for 16 h at 37°C and 200 rpm in 50 mL flasks. The bioreactors were inoculated with 8 mL of this pre-inoculum. Protein expression was induced using 0.55 mM IPTG for 4 h as of the end of the exponential growth phase (Abs600 nm of around 2.0). Samples were taken every hour to analyze cell growth, acetic acid concentration, L-asparaginase activity, and total protein produced. Protein expression was confirmed by enzyme activity analysis. Cell growth was evaluated by absorbance at 600 nm, and acetic acid concentration was evaluated by high-performance liquid chromatography.

### Enzymatic assay

Enzyme activity was measured by hydrolyzing asparagine to aspartate and ammonia [18]. One international unit (IU) of L-asparaginase was defined as the amount of enzyme capable of releasing 1 μmol of ammonia per minute at 37°C and pH 7.3 [8,19].

To analyze intracellular enzyme activity, we used the cells from 1 mL culture, to which 1 mL sodium phosphate buffer (0.02 M, pH 7.3) was added. This cell suspension was submitted to five 10-second cycles of sonication (30% amplitude—using a Sonics Vibra-Cell™ ultrasonic processor—VC 750), and was later used in the analysis of enzyme activity and quantification of total protein by absorbance at 595 nm, as described by Bradford (1976) [20], using a standard BSA curve. To analyze extracellular enzyme activity, 10 mL samples of cell-free medium were concentrated tenfold and the medium was replaced by a sodium phosphate buffer (0.02 M, pH 7.3) using ultrafiltration units with a 10 kDa membrane (Amicon Ultra-15, Millipore) and centrifuging at 4000 g for 30 minutes.

50 μL aliquots of these samples were added to 50 μL asparagine (5 g/L). This reaction was incubated at 37°C for 30 minutes, then 40 μL aliquots were immediately removed and added to 10 μL trichloroacetic acid (1.5 M). 2 mL of a reagent composed of sodium salicylate (60 mM), sodium nitroprusside (3.4 mM), and disodium EDTA (1.35 mM) was then added, and the mixtures were agitated in a vortex equipment. Next, 2 mL of a second reagent composed of sodium hypochlorite (4.8 mM) and sodium hydroxide (150 mM) was added, since the ammonium ions present in these reagents form a blue-green chromogenic compound [21]. The mixture was incubated at 37°C for 5 minutes. The sample was then analyzed for absorbance at 600 nm. The concentrations of ammonium ions released in the enzyme reaction were determined from a standard curve.

### E. coli cell growth measurement

Cell growth was measured by absorbance at 600 nm using a Shimadzu spectrophotometer (UV Mini 1240 UV/VIS) and cell concentration was expressed in dry cell weight. One unit of Abs600 nm corresponded to 2.82 g dry cell weight.L^-1^.

### Cell line

The Reh cell line (ETV6/RUNX1 [+]), a B cell precursor leukemia cell line with translocation (12;21) (ATCC CRL-8286) [22, 23], was used as a model for evaluating the antileukemic effects of recombinant L-asparaginase from *Z*. *mobilis*. This cell line was acquired from the Rio de Janeiro Cell Bank, Xerém, Duque de Caxias, RJ, Brazil. It was cultured in RPMI 1640 (Sigma-Aldrich, St. Louis, MO, USA), supplemented with 10% fetal bovine serum (Cultilab, Campinas, SP, Brazil), 100 μg/mL streptomycin, 100 IU/mL penicillin, and 2 mmol/L L-glutamin (Sigma-Aldrich, St. Louis, MO, USA) in a humidified incubator with 5% CO_2_ at 37°C. The Reh cell line was monitored constantly by both karyotype and FISH evaluations to detect ETV6-RUNX1 gene fusion. All cell culture experiments were initiated with 1x10^6^ cells/mL in the controls and treated samples.

### Patient cell samples

Bone marrow cells and peripheral blood cells were obtained from leftover material harvested for the diagnosis of four patients with ALL (mean age: 4.75 years old; range: 1–9 years old). Mononuclear cells were isolated by centrifugation on a Ficoll-Paque gradient (GE Healthcare Life Sciences, New Jersey, USA). Cells were plated in RPMI 1640 medium (Sigma-Aldrich, St. Louis, MO, USA) supplemented with 20% fetal bovine serum (Cultilab, Campinas, SP, Brazil), 100 μg/mL streptomycin, 100 IU/mL penicillin, and 2 mmol/L L-glutamine (Sigma-Aldrich, St. Louis, MO, USA).

### Cell treatment with extracellular recombinant L-asparaginase

The Reh cell line and primary cells from patients at 1×10^6^/mL were treated with different concentrations of extracellular recombinant L-asparaginase from *Z*. *mobilis* and incubated for 48 h in a humidified incubator with 5% CO_2_ at 37°C. The cell-free supernatant containing the extracellular enzyme produced in *E*. *coli* BL21 (DE3)/pET26b/*ans* was concentrated by ultrafiltration. The concentrated material was washed with 0.02 M sodium phosphate buffer (pH 7.3) and resuspended in mannitol solution (80 mg mannitol/10.000 UI) for application in the cell culture.

Reh cells were also treated with one of the commercially available forms of *E*. *coli* L-asparaginase (Elspar^®^). This enzyme was diluted in RPMI medium, and the manufacturer’s data on enzyme activity were used to calculate the dilution necessary for the doses used.

Controls were made with untreated cells (untreated control) or treated cells with samples derived from a growth process with the same genetically modified *E*. *coli* without the addition of IPTG, i.e., without inducing the expression of recombinant L-asparaginase from *Z*. *mobilis* (negative control). Enzymatic activity was also measured in the negative control for evaluating background activity from endogenous asparaginases from *E*. *coli*.

### Cell proliferation assay

Cells were cultured in the absence or presence of increasing concentrations of recombinant L-asparaginase from *Z*. *mobilis* for 48 h. Cell viability was determined using 3-(4,5-dimethylthiazol-2-yl)-2,5-diphenyltetrazolium bromide (MTT, Sigma-Aldrich, St. Louis, MO, USA) according to the manufacturer’s instructions. The inhibitory rate was calculated as follows: inhibitory rate (%) = [(A−B)/A] ×100, where A is the mean absorbance at 570 nm of control cells and B is the mean absorbance of test sample cells at the same wavelength.

### Cell viability, apoptosis, and cell cycle analysis by flow cytometry

Total cell number and cell viability were established by propidium iodide (PI, Sigma-Aldrich, St. Louis, MO, USA) staining, according to the manufacturer’s instructions. To assess the percentage of apoptotic cells, flow cytometry was used after staining with Annexin V-FITC and PI (Annexin V-FITC Apoptosis Detection Kit I, BD Biosciences, San José, CA), according to the manufacturer’s instructions. After staining with PI and Annexin V, the Perfect-Count Microspheres^™^ (Cytognos, Salamanca, Spain) was added in a 1:1 ratio (v/v), according to the manufacturer's recommendations for cell quantification. Following, at least 20,000 events were acquired per test in a FACSCanto II flow cytometer (BD, San José, CA, USA), using FACSDiVa software. Data were analyzed using Infinicyt software (Cytognos SL, Salamanca, Spain). After doublets exclusion in a forward scatter (FCS)-H *vs*. FCS-A dot plot, the cell populations were identified according Annexin and PI expression: viable cells (Annexin^-^ PI^-^), cells in early apoptosis (Annexin^+^ PI^-^), cells in late apoptosis (Annexin^+^ PI^+^), and necrotic cells (Annexin^-^ PI^+^). Beads were identified being two populations of side scatter (SSC)^hi^/FSC^lo^ events [24]. The experiments were considered adequate for cell quantification if the ratio between these two beads groups was 1:1. After that, we quantify the events in a gate containing the two beads populations and in each cell population. Following, the concentration of each population cells was calculated using the formula: number (n) of cells/μL = n of cell events/(n of microsphere events *x* n of beads/μL). Cell cycle was evaluated using Cycloscope^™^ (Cytognos SL, Salamanca, Spain) according to the manufacturer’s instructions.

### Nucleus morphology using DAPI staining evaluated in a fluorescence microscopy

Aliquots of 100 μL were washed in PBS buffer solution and then fixed in methanol and acetic acid (3:1) and stained with 0.125 μg/mL DAPI solution (4',6-diamydine-phenylindole, Cytocell) to observe the morphological alterations of the nuclei. The nucleus count was done using an Olympus BX51 microscope with a 100 W mercury-vapor lamp(Olympus, Miami, FL, USA) and a U-MWU2 filter with a 100x objective lens.

### Data statistical analysis of cytotoxicity

The results are reported as the median ± standard deviation of triplicate experiments. Statistical significance was accessed using the nonparametric Mann-Whitney test. A *p*-value ≤ 0.05 was considered statistically significant. All analyses were performed with SPSS IBM software, version 20 (San Diego, CA, USA).

### Ethics statement

The study was approved by the Research Ethics Committee of Instituto de Puericultura e Pediatria Martagão Gesteira, Federal University of Rio de Janeiro (IPPMG/UFRJ). Bone marrow cells and peripheral blood cells were obtained from leftover material harvested for the diagnosis of a patient with ALL. These samples were used after the patient’s parents had signed an informed consent form, as required by the Research Ethics Committee and recommended by the Declaration of Helsinki.

## Results and Discussion

### *In silico* characterization of L-asparaginase from Z. mobilis

The molecular mass and size of L-asparaginase from *Z*. *mobilis* (ATCC 31821) were estimated *in silico* as being 366 aa and 38.7 kDa, respectively, while the protein’s theoretical pI was predicted as 6.18. By ascertaining the presence of possible signal sequences in the enzyme’s amino acid sequence, we were able to infer that the first 29 amino acids are very likely to be part of a signal peptide. This is consistent with preliminary results with the enzyme [15], which demonstrated that L-asparaginase from *Z*. *mobilis* is a periplasmic enzyme. Other L-asparaginases have a signal peptide, like the type II periplasmic enzyme from *E*. *coli*, which has antileukemic activity [25–27], and the enzyme from *E*. *chrysanthemi*, which is also used to treat ALL [5,27]. Meanwhile, the type I L-asparaginase from *E*. *coli*, which does not have antileukemic activity, does not have a signal peptide because it is a cytoplasmic enzyme [25,27].

**Fig 2:**
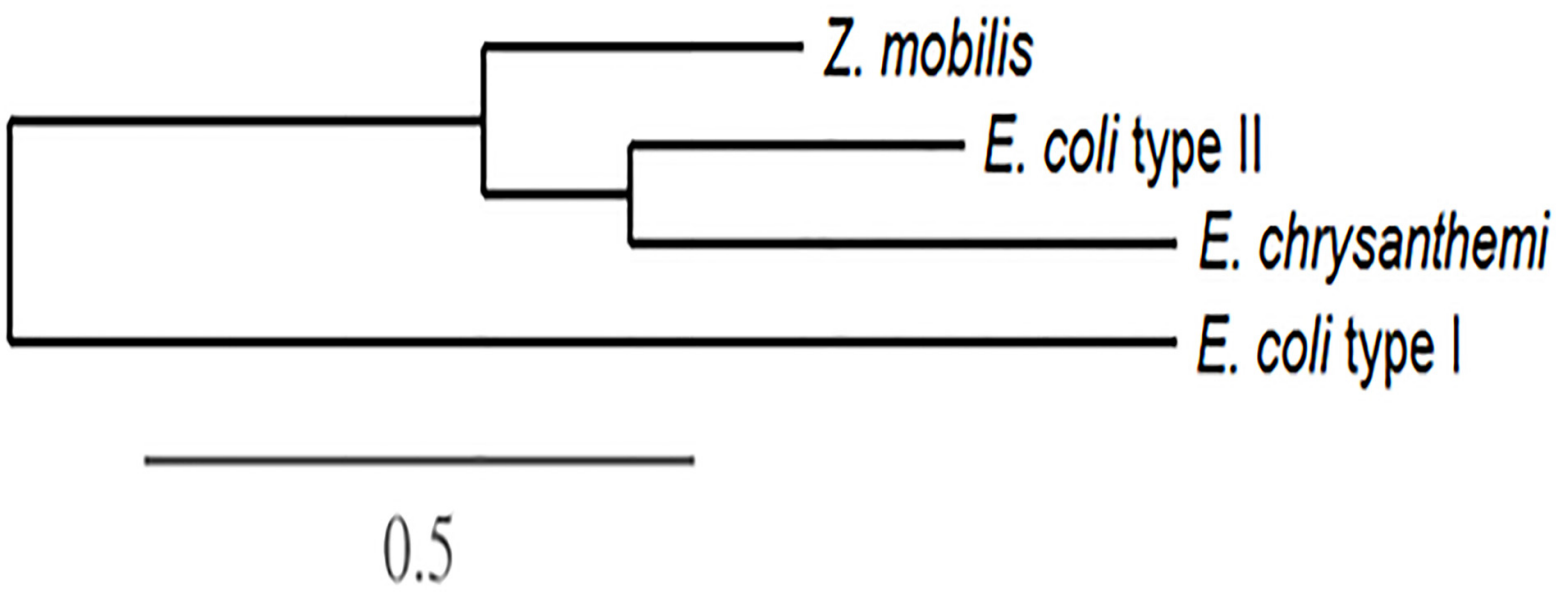
Phylogenetic tree of the different L-asparaginases from *Z*. *mobilis*, type II from *E*. *coli* and *E*. *chrysanthemi*, and type I from *E*. *coli*. Note the grouping of the enzyme from *Z*. *mobilis* with the type II enzymes from *E*. *coli* and *E*. *chrysanthemi*, both of which have antileukemic activity.

For the preliminary study of the possible antineoplastic activity of L-asparaginase from *Z*. *mobilis*, its nucleotide sequence was aligned with the nucleotide sequences of the L-asparaginases from *E*. *coli* and from *E*. *chrysanthemi*, since they both have antineoplastic activity and are used in commercial formulations. Global alignments were also made between the amino acid sequences of type I and type II L-asparaginases from *E*. *coli*, *E*. *chrysanthemi*, and *Z*. *mobilis*, and a phylogenetic tree was generated (Fig 2). This shows a grouping of the enzymes from *Z*. *mobilis* with the type II enzymes from *E*. *coli* and *E*. *chrysanthemi*. The sequence of the enzyme from *Z*. *mobilis* was found to show high identity to the sequence of the type II enzyme from *E*. *coli*– 40.4%–which is in the same percentage range as the identity between the proteins from *E*. *coli* and *E*. *chrysanthemi*, both of which have antileukemic activity. Meanwhile, the alignment between the two L-asparaginases from *E*. *coli* (type I, without antileukemic activity, and type II, with antileukemic activity) showed just 20.0% identity. These data could be seen as a further indication of the antileukemic activity of the enzyme from *Z*. *mobilis*.

Some studies in the literature present data on the alignment of new sequences of L-asparaginase that have cytotoxic effects on leukemia cells with sequences of commercial L-asparaginases from *E*. *coli* and *E*. *chrysanthemi*. L-asparaginase from *Pyrococcus furiosus* shows 22% identity to type II L-asparaginase from *E*. *coli*, but the authors do not present data on its alignment with L-asparaginase from *E*. *chrysanthemi* [28]. Meanwhile, L-asparaginase from *Helicobacter pylori* CCUG 17874 shows 18.8% identity with the sequence of type II L-asparaginase from *E*. *coli*, but 46.7% identity with the sequence of L-asparaginase from *E*. *chrysanthemi* [29].

Another indication of the antileukemic activity of the enzyme from *Z*. *mobilis* is that the amino acids in the active site of type II L-asparaginase from *E*. *coli* are highly conserved and aligned with the amino acids of the L-asparaginase from *Z*. *mobilis*. The amino acids from both catalytic triads (Thr-12, Tyr-25, Glu-283) and (Thr-89, Lys-162, Asp-90) are conserved and aligned with the sequences of the type II L-asparaginases with known antileukemic activity (*E*. *coli* and *E*. *chrysanthemi*) and the amino acid sequence of the type II enzyme of *Z*. *mobilis* (these alignments are shown in S1 Supplementary Material).

### Cloning and expression of L-asparaginase

The gene of L-asparaginase from *Z*. *mobilis* used in this study was chemically synthesized, and fused to a histidine tag. The codons were optimized to the codons most frequently used in *E*. *coli* (modifying codons with a frequency of less than 15%) and to prevent strong secondary structures (modifying codons to this end).

In order to verify the expression of cytoplasmic and extracellular L-asparaginase, the recombinant *E*. *coli* with the L-asparaginase gene of interest was cultivated and expressed in bioreactors using LB medium supplemented with glucose. Both the clones obtained in this study, *E*. *coli* BL21(DE3)/pET26b/*ans* (extracellular enzyme) and *E*. *coli* BL21(DE3)/pET28a/*ans* (cytoplasmic enzyme), were used. The cultures were induced with 0.55mM IPTG for 4 h, when they attained absorbance of around 2.0.

**Fig 3:**
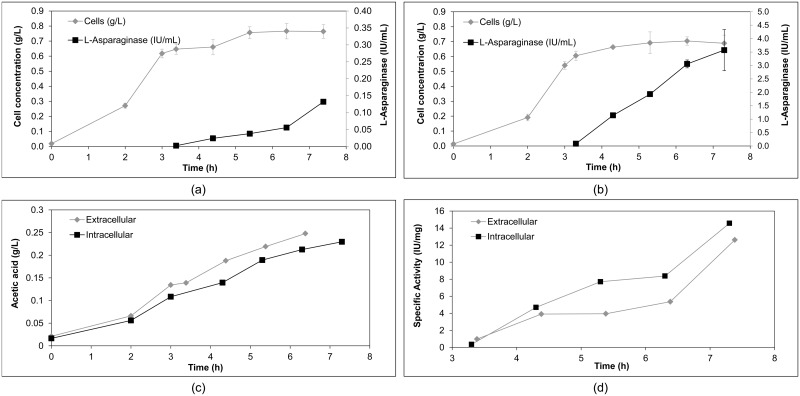
(a) Cell growth and activity of L-asparaginase using *E*. *coli* BL21 (DE3)/pET26b/*ans* (extracellular expression) induced after 203 minutes of culture; (b) growth and expression of the protein using *E*. *coli* BL21 (DE3)/pET28a/*ans* (intracellular expression) induced after 198 minutes of culture; (c) production of acetic acid over time; (d) specific activity of L-asparaginase over time as of induction of protein expression using the recombinant strains of L-asparaginase (*E*. *coli* BL21 (DE3)/pET26b/*ans* and *E*. *coli* BL21 (DE3)/pET28a/*ans*). The results are the mean of the data, with error bars representing the standard deviation of triplicate values.

Cell growth and L-asparaginase expressed and released by *E*. *coli* BL21 (DE3)/pET26b/*ans* throughout the growth period are shown in Fig 3(a). After around 7 h (420 min) culture time, a cell concentration of 0.76 g/L from the *E*. *coli* BL21(DE3)/pET26b/*ans* strain was obtained. Activity of 0.132 IU/mL extracellular enzyme and a maximum yield of 172.7 IU/g dry cell weight were obtained after 4 h induction of expression (Table 1).

**Table 1:** Cell concentration and enzyme activity of the cultures in bioreactors in LB medium at 37°C after 4 h induction with 0.55 mM IPTG.

Strain	DCW (g/L)	Activity (IU/mL)	Yp/x (IU/g dry cell weight)	Productivity (IU/mg protein.h culture)
*E*. *coli* BL21 (DE3)/pET26b/*ans* (extracellular expression)	0.76	0.13	173	1.7
*E*. *coli* BL21 (DE3)/pET28a/*ans* (intracellular expression)	0.7	3.6	5186	2.0

DCW = dry cell weight; Yp/x = yield per gram of cell.

Khushoo et al. (2005) [30] investigated the extracellular expression of L-asparaginase from *E*. *coli* K-12 in *E*. *coli* BLR (DE3) in bioreactors using TB (Terrific Broth) as a culture medium in fed batches. After 35 h cultivation, in 24 h of which expression was induced, these authors obtained cell concentration of 45 g/L and activity of 870 IU/mL, with 19333 IU/g dry cell weight [30]. These values are higher than those obtained in the experiments presented in Fig 3(a) and Table 1, because in their work, Khushoo *et al*. (2005) [30] employed much longer cultivation periods (35 h) and used a fed-batch system, which enabled higher cell growth and protein formation, unlike the batch system used in this study. We should add that comparisons between the expression of other L-asparaginases and the L-asparaginase produced here through enzyme activity are unreliable because activity analyses are conducted under different temperatures and pHs, and using different methods.

Cell growth and L-asparaginase expressed in the cytoplasm by *E*. *coli* BL21 (DE3)/pET28a/*ans* throughout the culture time is shown in Fig 3(b) and Table 1. After around 7 h (420 min) it was possible to obtain a cell concentration of 0.68 g/L for *E*. *coli* BL21(DE3)/pET28a/*ans*, which is similar to the concentration obtained for the strain with extracellular expression. After 4 h induction, 3.57 IU/mL culture medium was obtained (intracellular enzyme), with a maximum yield of 5185 IU/g dry cell weight after 4 h induction, which means the yield was far higher than that of extracellular expression.

The L-asparaginase production levels presented in this study are higher than those obtained using the native microorganism of L-asparaginase (*Z*. *mobilis*). In cultures of *Z*. *mobilis* in shaking flasks, the activity level obtained was 37.8 IU/g dry cell weight after 33 h culture [15]. By producing recombinant L-asparaginase, around four times higher IU/g dry cell weight of the extracellular enzyme was obtained, and 135 times higher IU /g dry cell weight of the intracellular enzyme in a quarter of the time.

In these experiments, we also analyzed acetic acid concentration, since this can inhibit cell growth. Some authors have found that inhibition occurs when acetic acid exceeds 2 g/L [31], while others suggest it occurs when acetic acid exceeds 5 g/L [32], and others have found that protein expression could be hampered by far lower values, of 0.9 to 1.5 g/L [33]. The acetic acid concentrations obtained in the analyses are shown in Fig 3(c), where it can be seen that the concentration was around 0.25 g/L by the end of the culture time for both strains, which is far below any of the values cited in the literature as inhibiting cell growth.

Although intracellular expression was higher, when we analyzed the values in terms of specific activity (i.e. L-asparaginase activity per total protein) we found that the L-asparaginases expressed extracellularly and intracellularly demonstrated similar specific activity (Fig 3(d)). As a larger quantity of L-asparaginase was produced intracellularly than extracellularly but their specific activity was similar, it is fair to say that the sample of the extracellular protein contained fewer contaminants, which would greatly facilitate its purification. Furthermore, the use of a process where the product is obtained extracellularly rules out the need for the cell disruption stage, reducing production time and costs. For these reasons, the *E*. *coli* BL21 (DE3)/pET26b/*ans* clone (extracellular expression) was chosen to be used in the tests on cells from patients with ALL.

### Cytotoxicity analysis of L-asparaginase on cell line

**Fig 4:**
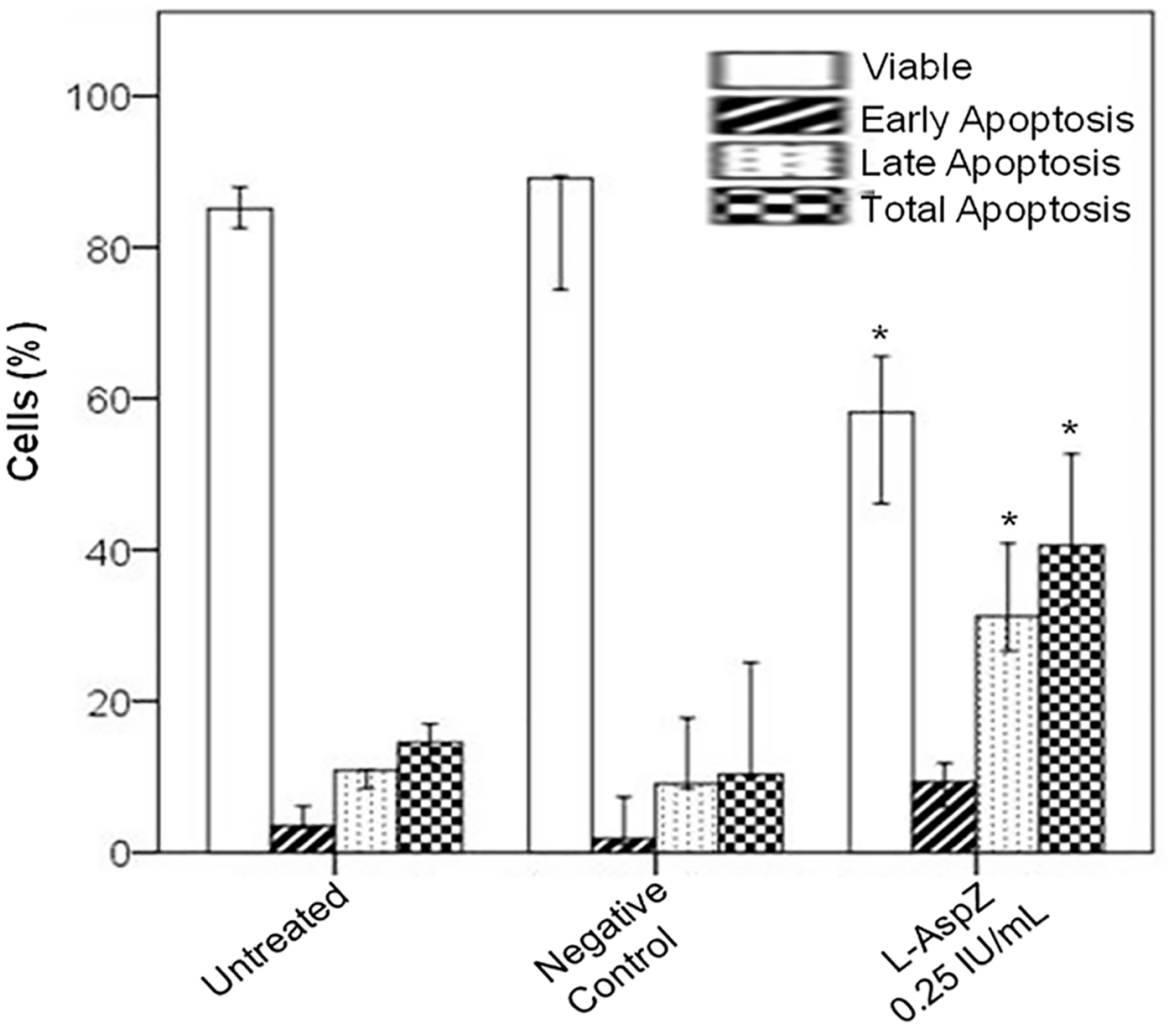
Apoptotic effect of recombinant L-asparaginase from *Z*. *mobilis* on Reh cell line. Cells were plated in triplicate and treated with recombinant L-asparaginase from *Z*. *mobilis* (L-AspZ) 0.25 IU/mL. They were incubated with Annexin V FITC and PI and analyzed by flow cytometry. Data represent the L-AspZ-induced increase in apoptotic cells, compared to the respective values observed in untreated control cultures and negative control cultures. The results are the median of the data, with error bars representing the standard deviation of triplicate values. *p ≤ 0.05.

The Reh cell line was used to measure the cytotoxic effect of the extracellular preparation of L-asparaginase from *Z*. *mobilis*. Cells were treated with L-asparaginase from *Z*. *mobilis*. (0.25 IU/mL). The untreated control and negative control were made. Cells were analyzed by flow cytometry 48 h after treatment. L-asparaginase from *Z*. *mobilis* 0.25 IU/mL markedly reduced the total number of cells, and this dose caused an increase in the percentage of apoptotic Reh cells. As in other studies investigating the sensitivity of L-asparaginase, a reduction in the concentration of viable cells was observed in the samples treated with the enzyme [34–36] (Table 2). L-asparaginase from *Z*. *mobilis* also induced apoptosis in Reh cells (Fig 4). Apoptosis seems to be a key indicator of cellular response to cytotoxic drugs. Like other studies evaluating apoptosis in Reh cell line, staining with Annexin V FITC was used to identify the phosphatidylserine exposure that occurs in cell death by apoptosis [35, 37]. Thus, the cells stained only with Annexin V FITC corresponded to the percentage of cells in early apoptosis, whereas cells stained with Annexin V FITC and PI corresponded to the percentage of cells in late apoptosis. Our results clearly showed that Annexin V FITC stained the cells treated with recombinant L-asparaginase from *Z*. *mobilis*, suggesting that apoptosis was induced by the action of the enzyme. These data are consistent with the proposed mechanism of action of this enzyme.

**Table 2:** Cell concentration and percentage of viable cells after treatment of Reh cells with recombinant L-asparaginase from *Z*. *mobilis*. The cells were cultured in the presence and absence (control) of L-asparaginase 0.25 IU/mL and in the presence of a control without enzyme induction (negative control). Cell concentration and percentage of viable cells were determined by staining with PI and flow cytometry 48 h after treatment. The data are the mean ± standard deviation of the experiments in triplicate.

Treatment	Cell Concentration (no. of cells per mL)	% Viable Cells
Control	5.57 x 10^6^ ± 2.25 x 10^6^	85.2 ± 2.7
Negative Control	5.54 x 10^6^ ± 1.51 x 10^6^	84.3 ± 8.5
*E*. *coli* BL21 (DE3)/pET26b/*ans* (0.25 IU/mL)	5.87 x 10^5^* ± 1.83 x 10^5^	56.6* ± 9.8

*p ≤ 0.05

The results of the treatment with recombinant L-asparaginase from *Z*. *mobilis* at 0.25 UI/mL were compatible with clinical studies *in vivo* that suggest that the level of L-asparaginase should be between 0.1 UI/mL and 0.4 UI /mL to obtain the L-asparagine depletion required for therapeutic benefit [38–45]. In addition, our finding is even more relevant when compared to a study *in vitro* that simulated the BFM protocol for L-asparaginase dosage, which evaluated the response on Reh cells using doses of 0.4 UI/mL and 4.0 UI/mL, since the protein extract containing enzyme from *Z*. *mobilis* was able to show effects at a dose with lower enzyme activity [46].

**Fig 5:**
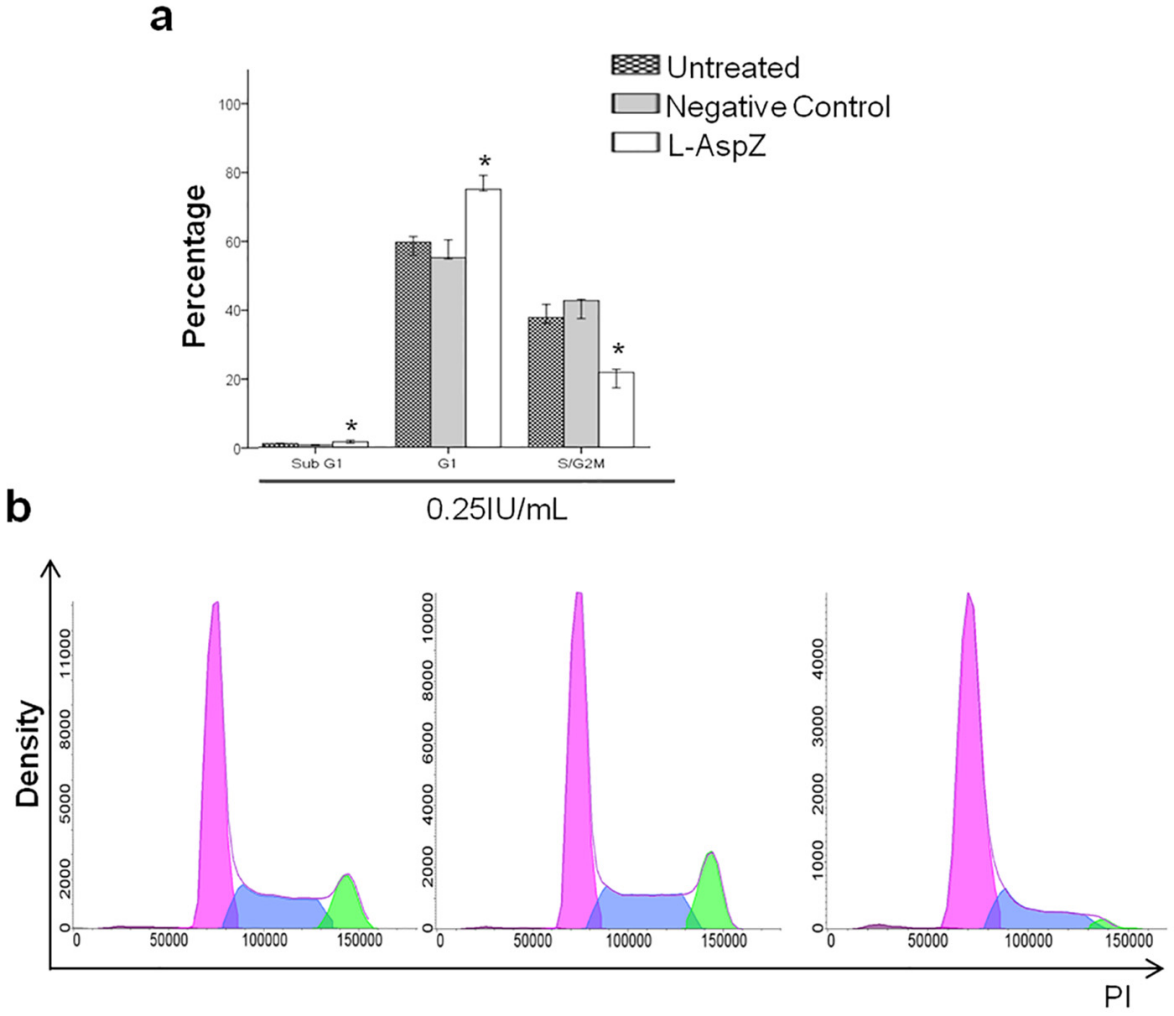
Effect of recombinant L-asparaginase from *Z*. *mobilis* on cell cycle distribution of Reh cell line. Reh cells were treated with 0.25 IU/mL of L-asparaginase from *Z*. *mobilis* (L-AspZ), compared to the respective values observed in untreated control cultures and negative control cultures. The cells were stained with PI and DNA content was analyzed by flow cytometry. **(a)** Results are the median of data, with error bars representing the standard deviation of triplicate values. **(b)** The representative results of three independent experiments are shown. *p ≤ 0.05.

Since previous reports on *in vitro* studies have found that L-asparaginase causes cell cycle arrest [47], the effect of the enzyme from *Z*. *mobilis* on Reh cell cycle status was examined. Reh cells showed a significant accumulation in the G0/G1 phase (Fig 5) (*p* ≤ 0.05). The same was observed in another study *in vitro*, where cell lines treated with L-asparaginase showed a significant increase in the number of cells in the G1 phase and a decrease in the number of cells in the S phase[47,48]. In addition, in a study *in vitro* that evaluated asparaginase from *Enterobacter cloacae* against cell lines of human cancer, the cell cycle progression analysis indicated that the enzyme induced apoptosis by cell cycle arrest at the G0/G1 phase [49]. Our results are in agreement with previous reports about the mechanism of action proposed for L-asparaginase, since it has been demonstrated that L-asparaginase causes cell cycle arrest at G0/G1 and loss of viability due to apoptosis [45–47]. It is known that the enzymatic activity of L-asparaginase also causes L-glutamine depletion and studies have demonstrated that the concentration of L-glutamine is important in the progression to the S phase of the cell cycle in various cells [50–52]. Our data showed the arrest of the cell cycle at the G1 phase. However, it was not possible to correlate the effects observed with biochemical characteristics, such as the L-glutaminase activity of recombinant L-asparaginase from *Z*. *mobilis*, because this feature was not tested.

**Fig 6:**
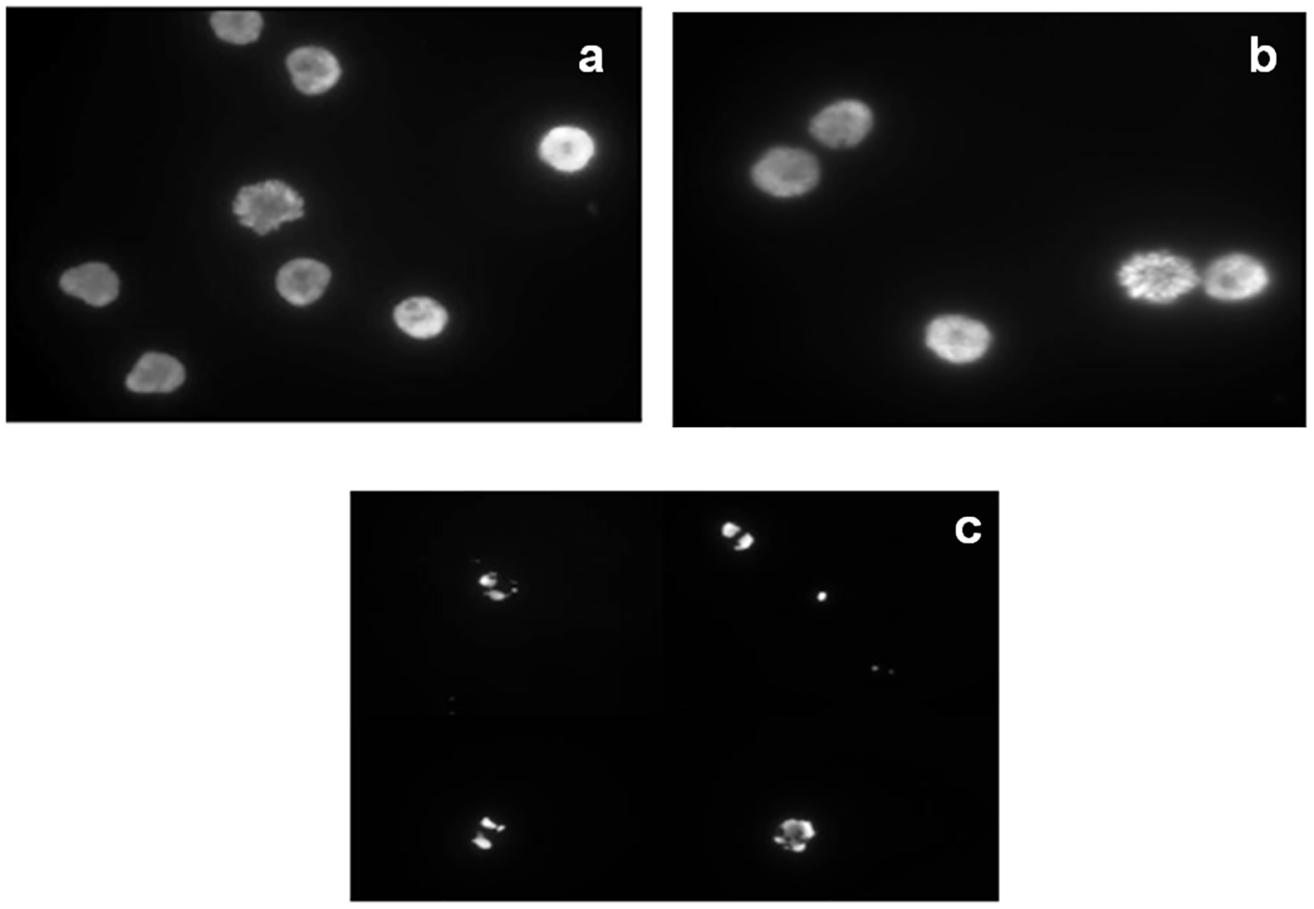
Alteration in nuclear morphology by treatment with protein extract containing recombinant L-asparaginase from *Z*. *mobilis* after DAPI staining. Representative images of Reh cells in fluorescence microscopy (1000x), **(a)** untreated control; **(b)** negative control and **(c)** the sample treated with protein extract containing recombinant L-asparaginase from *Z*. *mobilis*.

As expected, the cells of the untreated control and negative control showed similar profiles, with a higher number of viable cells and lower percentage of apoptosis compared to cells treated with L-asparaginase from *Z*. *mobilis*. The untreated control and negative control experiments yielded similar results, with statistically significant differences compared to the treated samples (Fig 4) (*p* = 0.05). These results were also confirmed by the presence of apoptotic nuclei in the samples treated with the extract containing L-asparaginase from *Z*. *mobilis* (Fig 6). The morphological evaluation was conducted to observe the apoptotic nuclei. A higher number of apoptotic nuclei in the treated samples was observed, while a higher number of normal nuclei and also the presence of mitotic nuclei were observed in the untreated control samples and negative control samples (Fig 6(a), 6(b) and 6(c)). Microscopy was used as an additional method for qualitative analysis in order to view the apoptotic nuclei and confirm the results obtained by flow cytometry.

The results obtained after treatment of the Reh cells with the negative control, where the cells were treated with a sample without the induction of recombinant L-asparaginase expression, suggest that the cytotoxic effects observed in the treated cells could be attributed to the presence of the L-asparaginase enzyme from *Z*. *mobilis* and not some component from the medium. Also, no enzymatic activity was found in the negative control performed without inducing the expression of recombinant L-asparaginase from *Z*. *mobilis*, indicating that no background activity from the host endogenous asparaginases was present.

**Fig 7:**
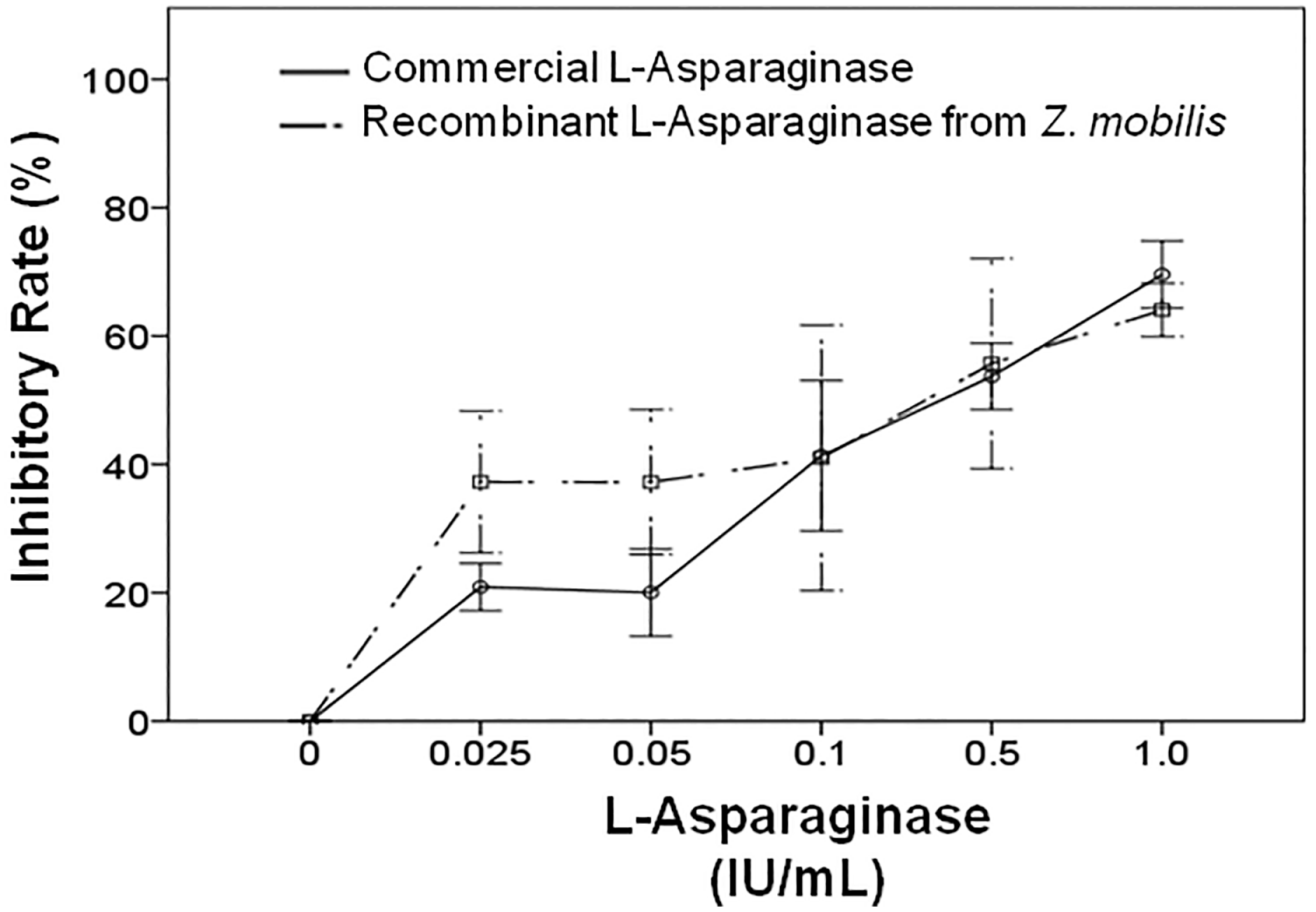
Reduced proliferation of Reh cell line induced by Recombinant L-asparaginase from *Zymomonas mobilis* and commercial L-asparaginase. Reh cells were treated with Recombinant L-asparaginase from *Zymomonas mobilis* and commercial L-asparaginase (0.00–1.00 IU/mL) and evaluated using the MTT assay. Data represent the L-asp-induced decrease in the percentage of viable cells compared to the respective values observed in control cultures. Results are the median of data, with error bars representing the standard deviation of triplicate values.

Recombinant L-asparaginase and commercial L-asparaginase significantly inhibited the proliferation of Reh cells in a dose-dependent manner (Fig 7). By comparing the effect of recombinant L-asparaginase from *Z*. *mobilis* and commercial L-asparaginase on Reh cells, it was found that the enzymes showed different results when lower doses were used. At doses lower than 0.1 IU/mL, commercial L-asparaginase resulted in greater growth inhibition (*p* = 0.05). From the 0.1 IU/mL dose, which corresponds to the recommended dose for effective treatment with L-asparaginase in current protocols, both enzymes showed very similar results [43, 45]. These data suggest that it is possible to obtain equally effective results with the use of recombinant L-asparaginase from *Z*. *mobilis* at a dose range of 0.1–1.0 IU/mL.

### Cytotoxicity analysis of L-asparaginase on patient cell samples

**Fig 8:**
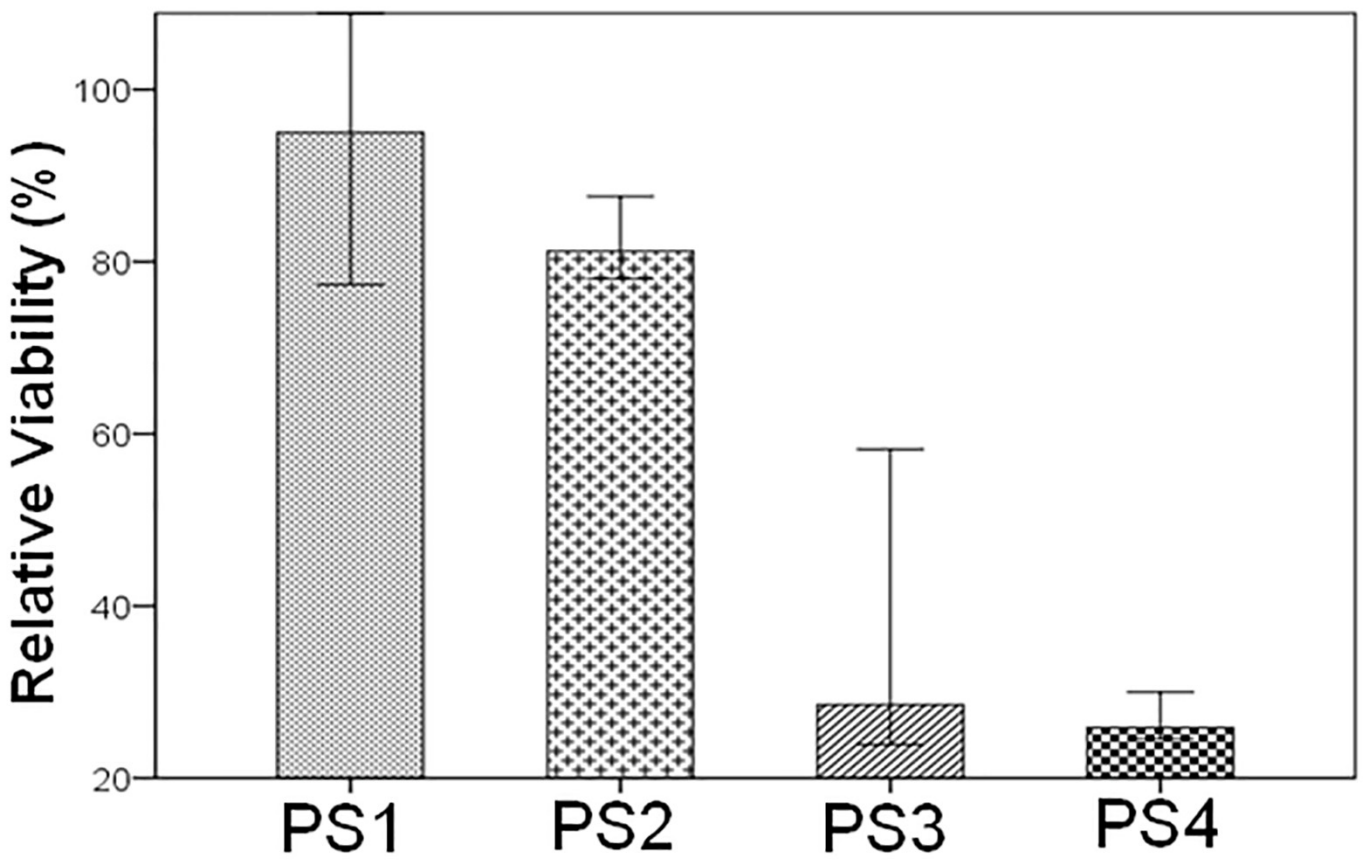
Effect of recombinant L-asparaginase from *Z*. *mobilis* on viability of ALL primary cells. Cells were treated with recombinant L-asparaginase from *Z*. *mobilis* 0.1 IU/mL Viability was analyzed by flow cytometry 48h after treatment. Data represent the percentage viability of the treated cells relative to the respective values observed in parallel untreated control cultures. Results are the median of the data, with error bars representing the standard deviation of triplicate values.

The effect of L-asparaginase from *Z*. *mobilis* on the 0.1 IU/mL dose was tested against four different ALL patient cell samples (whose clinical and laboratory characteristics are presented in Table 3). The primary samples showed different viability results after L-asparaginase treatment (Fig 8). Samples PS3 and PS4 showed significant cell viability reduction after treatment with the enzyme. In sample PS2, which had an isochromosome of the long arm of chromosome 7, although the percentage of viable cells was not low at the time of analysis, a peak corresponding to apoptotic cells was observed. This suggests that initially the action of L-asparaginase induced the apoptosis of these cells but at some time, due to loss of enzyme activity or cellular mechanisms, the cells were able to remain viable. Sample PS1, with t(1;19), E2A-PBX1 fusion gene, showed low responsiveness to the dose of L-asparaginase applied. Sample PS4, with hyperdiploidy, demonstrated a better response to treatment with L-asparaginase from *Z*. *mobilis*, resulting in significantly reduced cell viability. This result is in agreement with the high sensitivity to L-asparaginase of hyperdiploid cells, as reported by Krejci (2004) [46]. These results suggest the cytotoxicity of the recombinant L-asparaginase from *Z*. *mobilis* to leukemic cells, although the response to L-asparaginase may vary on different cell subtypes with different genetic characteristics. [45,53,54]

**Table 3:** Clinical and laboratory characteristics of ALL patients.

Sample	Age (years)	Sex (M/F)	BM/PB	WBC	% Blasts	Phenotype	Chromosomal Aberration
PS1	5	F	BM	111300	64	Common	t(1;19)
PS2	9	F	BM	16400	91	Common	+i(7)(q10)
PS3	1	M	PB	110000	65	Common	Not Evaluated
PS4	4	F	BM	50200	78	Pre-B	Hyperdiploid

Obs. The data refer to initial diagnosis parameters.

M = male; F = female; WBC = white blood cells (white cell count from hemogram—leucometry); samples from BM (bone marrow) or PB (peripheral blood); ALL phenotype classification from EGIL.

**Fig 9:**
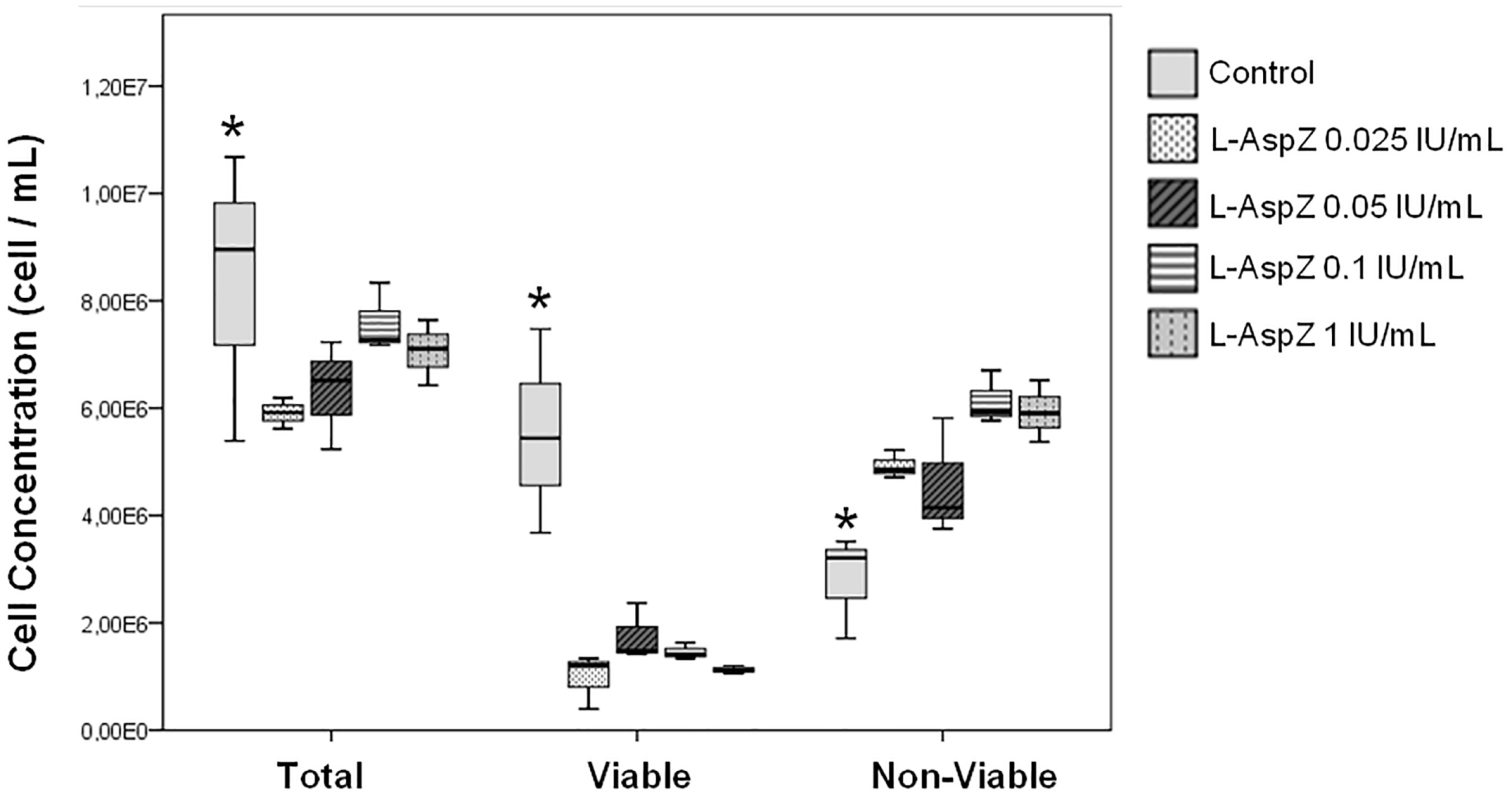
Cells from one ALL patient (PS4) treated with 0.0 IU/mL (control), 0.025 IU/mL, 0.05 IU/mL, 0.1 IU /mL, 0.5 IU/mL and 1.0 IU/mL recombinant L-asparaginase from *Z*. *mobilis* obtained from the *E*. *coli* BL21 (DE3)/pET26b/*ans* extracellular extract (L-AspZ) and stained with PI (X-axis). The box-plots represent the median values of the total cell concentration, viable cell concentration, and dead cell concentration observed 48 h after the addition of L-asparaginase. The error bars represent the standard deviation of the triplicates. *p ≤ 0.05.

Finally, the cytotoxicity of different doses of the extracellular preparation of L-asparaginase from *Z*. *mobilis* on sample PS4 was analyzed. The results show that the total concentration of cells and the concentration of viable cells was lower in the treated samples than in the control for all the doses tested (Fig 9). As in other studies of sensitivity to L-asparaginase using methods that specifically consider viable cells [34–36, 55], flow cytometry revealed a reduction in the concentration of viable cells in the samples treated with the L-asparaginase produced in *E*. *coli*. Statistically significant differences were observed between the treated samples and the control.

## Conclusions

The optimized gene of L-asparaginase from *Z*. *mobilis* was cloned in *E*. *coli* using vectors pET26b and pET28a. Two clones were obtained: *E*. *coli* BL21 (DE3)/pET26b/ans and *E*. *coli* BL21 (DE3)/pET28a/ans. Recombinant L-asparaginase was expressed extracellularly using *E*. *coli* BL21 (DE3)/pET26b/ans, yielding enzyme activity. Likewise, L-asparaginase was expressed using *E*. *coli* BL21 (DE3)/pET28a/ans, also yielding enzyme activity, but in this case the enzyme was obtained in the cytoplasm. This is the first time that the L-asparaginase from *Z*. *mobilis* has been produced recombinantly in a different host. Due to its technological potential, this innovation is the subject of a patent application filed in Brazil with Instituto Nacional de Propriedade Intelectual [56].

Higher production of L-asparaginase was obtained in this study than is obtained by the native microorganism of this L-asparaginase (*Z*. *mobilis*), whose low yield has hampered the study and production of the enzyme from this microorganism. By this method, it was possible to produce enough L-asparaginase to conduct antileukemic activity studies.

Although a higher intracellular than extracellular protein concentration was obtained, the specific activity values (L-asparaginase expressed per total protein) were similar. This suggests that the sample of extracellular protein contained fewer contaminants, facilitating its purification and ruling out the need for cell disruption, thereby reducing process time and costs. The recombinant L-asparaginase from *Z*. *mobilis* expressed extracellularly by the *E*. *coli* BL21(DE3)/pET26b/*ans* clone was evaluated in cytotoxicity tests on Reh cells and primary cells of patients with ALL, which indicated the potential antileukemic activity of the enzyme where cytotoxic activity was indeed detected.

Given the potential demonstrated by the protein extract containing recombinant L-asparaginase from *Z*. *mobilis* produced in *E*. *coli*, this work suggests that other characteristics of the enzyme should be investigated and improvements made to some of its biochemical features (e.g. purification, PEGylation, etc.) for its clinical application. With such improvements and a better understanding of its biochemical and pharmacological properties, it could become an additional option for the treatment of ALL in children.

## Supporting Information

S1 Supplementary Material (DOC)
