# Correction: Recombinant L-Asparaginase from *Zymomonas mobilis*: A Potential New Antileukemic Agent Produced in *Escherichia coli*

**DOI:** 10.1371/journal.pone.0163203

**Published:** 2016-09-14

**Authors:** Karen Einsfeldt, Isis Cavalcante Baptista, Juliana Christina Castanheira Vicente Pereira, Roberta Eitler Bruno, Fabiana Vieira Mello, Isabele Campos Costa-Amaral, Elaine Sobral da Costa, Maria Cecília Menks Ribeiro, Marcelo Gerardin Poirot Land, Tito Lívio Moitinho Alves, Ariane Leites Larentis, Rodrigo Volcan Almeida

Dr. Roberta Eitler Bruno and Dr. Fabiana Vieira Mello should be included in the author byline. They should be listed as the fourth and fifth authors respectively. Both authors are affiliated with number 3: Programa de Pós-Graduação em Clínica Médica, Universidade Federal do Rio de Janeiro (UFRJ), Rio de Janeiro, Brazil.

The correct citation is: Einsfeldt K, Baptista IC, Pereira JCCV, Bruno RE, Mello FV, Costa-Amaral IC, et al. (2016) Recombinant L-Asparaginase from *Zymomonas mobilis*: A Potential New Antileukemic Agent Produced in *Escherichia coli*. PLoS ONE 11(6): e0156692. doi:10.1371/journal.pone.0156692
