# Supplementary material for: Recombinant L-Asparaginase from Zymomonas mobilis: A Potential New Antileukemic Agent Produced in Escherichia coli
Source: PLoS One. 2016 Jun 2;11(6):e0156692. doi: 10.1371/journal.pone.0156692 (PMC4890785; doi:10.1371/journal.pone.0156692)
Supplement: S1 Supplementary Material — (DOC) [file pone.0156692.s001.doc]

**Supplementary material**

Global sequence alignments performed with the help of the program ClustalW between the protein amino acid sequences of L-asparaginase type I and type II from *Escherichia coli*,
*Erwinia chrysanthemi* and *Zymomonas mobilis*. The identical amino acids are shown in red in the alignments, the strongly similar amino acids are shown in green, the weakly similar amino acids are shown in the blue and the different amino acids are shown in black. The amino acids present in the active sites of the L-asparaginase type II (ans B) from *E. coli* and *Z. mobilis* are highlighted in yellow.

Global alignment of the amino acid sequence of type I L-asparaginase (ans A) and type II
L-asparaginase (ans B) from *Escherichia coli*

10 20 30 40 50 60

| | | | | |

ansAxx0 ------MQKKSIYVAYTGGTIGMQ----RSEQGYIPVSGHLQRQLALMP--------EFH

ansBxx1 MEFFKKTALAALVMGFSGAALALPNITILATGGTIAGGGDSATKSNYTVGKVGVENLVNA

:: :.::*.::.: : * *. .*. :

Prim.cons. MEFFKK22222222222G222222NITI2222G2I222G2222222222GKVGVENL222

70 80 90 100 110 120

| | | | | |

ansAxx0 RPEMPDFT--IHEYTPLMDSSDMTPEDWQHIAEDIKAHYDDYDGFVILHGTDTMAYTASA

ansBxx1 VPQLKDIANVKGEQVVNIGSQDMNDNVWLTLAKKINTDCDKTDGFVITHGTDTMEETAYF

*:: *:: * . :.*.**. : * :*:.*::. *. ***** ********

Prim.cons. 2P222D22NV22E222222S2DM2222W222A22I2222D22DGFVI2HGTDTM22TA22

130 140 150 160 170 180

| | | | | |

ansAxx0 LSFMLENLGKPVIVTGSQIPLAELRSDGQINLLNALYVAANYPIN--EVTLFFNNRLYRG

ansBxx1 LDLTVK-CDKPVVMVGAMRPSTSMSADGPFNLYNAVVTAADKASANRGVLVVMNDTVLDG

*.: :: .***::.*: * :.: :** :** **: .**: . * :.:*: : *

Prim.cons. L22222N22KPV222G222P222222DG22NL2NA222AA22222NR2V2222N22222G

190 200 210 220 230 240

| | | | | |

ansAxx0 NRTTKAHADGFDAFASPNLPPLLEAG-IHIRRLNTPPAPHG-EGELIVHPITPQP-IGVV

ansBxx1 RDVTKTNTTDVATFKSVNYGPLGYIHNGKIDYQRTPARKHTSDTPFDVSKLNELPKVGIV

. .**::: .. :* * * ** :* .**. * : : * :. * :*:*

Prim.cons. 222TK22222222F2S2N22PL2222N22I2222TP222H2S22222V222222PK2G2V

250 260 270 280 290 300

| | | | | |

ansAxx0 TIYPGISADVVRNFLRQPVKALILRSYGVGNAPQNKAFLQELQEASDRGIVVVNLTQCMS

ansBxx1 YNYANASDLPAKALVDAGYDGIVS--AGVGNGNLYKSVFDTLATAAKTGTAVVRSSRVPT

*.. * .: :: ..:: ****. *:.:: * *:. * .**. :: :

Prim.cons. 22Y222S22222222222222222RS2GVGN2222K22222L22A222G22VV2222222

310 320 330 340 350 360

| | | | | |

ansAxx0 GKVNMGGYATGNALAHAGVIGGADMTVEATLTKLHYLLSQELDTETIRKAMSQNLRGELT

ansBxx1 GATTQDAEVDD---AKYGFVASGTLNPQKARVLLQLALTQTKDPQQIQQIFNQY------

* .. .. . . *: *.:... :. : : . *: *:* *.: *:: :.*

Prim.cons. G2222222222NALA22G222222222222222L222L2Q22D222I22222Q2LRGELT

ansAxx0 PDD

ansBxx1 ---

Prim.cons. PDD

Global alignment of amino acid sequences of type II L-asparaginase (ans B) from *Erwinia chrysanthemi* and type II L-asparaginase (ans B) from *Escherichia coli*

10 20 30 40 50 60

| | | | | |

AnsB_erwinia_chrysanthemi MERWFKSLFVLVLFFVFTASAADKLPNIVILATGGTIAGSAATGTQTTGYKAGALGVDTL

ansB_E_coli ME-FFKKTALAALVMGFSG-AALALPNITILATGGTIAGGGDSATKSN-YTVGKVGVENL

** :**. : .*.: *:. ******.**********..:.*::. *..* :**:.*

Prim.cons. MER2FK222222L222F22SAA22LPNI2ILATGGTIAG22222T222GY22G22GV22L

70 80 90 100 110 120

| | | | | |

AnsB_erwinia_chrysanthemi INAVPEVKKLANVKGEQFSNMASENMTGDVVLKLSQRVNELLARDDVDGVVITHGTDTVE

ansB_E_coli VNAVPQLKDIANVKGEQVVNIGSQDMNDNVWLTLAKKIN--TDCDKTDGFVITHGTDTME

:****::*.:*******. *:.*::*..:* *.*::::* *..**.********:*

Prim.cons. 2NAVP22K22ANVKGEQ22N22S22M222V2L2L2222NEL222D22DG2VITHGTDT2E

130 140 150 160 170 180

| | | | | |

AnsB_erwinia_chrysanthemi ESAYFLHLTVKSDKPVVFVAAMRPATAISADGPMNLLEAVRVAGDKQSRGRGVMVVLNDR

ansB_E_coli ETAYFLDLTVKCDKPVVMVGAMRPSTSMSADGPFNLYNAVVTAADKASANRGVLVVMNDT

*:****.****.*****:*.****:*::*****:** :** .*.** * .***:**:**

Prim.cons. E2AYFL2LTVK2DKPVV2V2AMRP2T22SADGP2NL22AV22A2DK2S22RGV2VV2ND2

190 200 210 220 230 240

| | | | | |

AnsB_erwinia_chrysanthemi IGSARYITKTNASTLDTFKANEEGYLGVIIGNRIYYQNRIDKLHTTRSVFDVRGLTSLPK

ansB_E_coli VLDGRDVTKTNTTDVATFKSVNYGPLGYIHNGKIDYQRTPARKHTSDTPFDVSKLNELPK

: ..* :****:: : ***: : * ** * ..:* **. : **: : *** *..***

Prim.cons. 2222R22TKTN22222TFK2222G2LG2I2222I2YQ222222HT2222FDV22L22LPK

250 260 270 280 290 300

| | | | | |

AnsB_erwinia_chrysanthemi VDILYGYQDDPEYLYDAAIQHGVKGIVYAGMGAGSVSVRGIAGMRKAMEKGVVVIRSTRT

ansB_E_coli VGIVYNYANASDLPAKALVDAGYDGIVSAGVGNGNLYKSVFDTLATAAKTGTAVVRSSRV

*.*:*.* : .: .* :: * .*** **:* *.: : : .* :.*..*:**:*.

Prim.cons. V2I2Y2Y222222222A2222G22GIV2AG2G2G222222222222A222G22V2RS2R2

310 320 330 340 350

| | | | |

AnsB_erwinia_chrysanthemi GNGIVPPDEELPG-----LVSDSLNPAHARILLMLALTRTSDPKVIQEYFHTY

ansB_E_coli PTGATTQDAEVDDAKYGFVASGTLNPQKARVLLQLALTQTKDPQQIQQIFNQY

.* .. * *: .:.*.:*** :**:** ****:*.**: **: *: *

Prim.cons. 22G2222D2E222AKYGF22S22LNP22AR2LL2LALT2T2DP22IQ22F22Y

Global alignment of amino acid sequences of type II L-asparaginase (ans B) from *Zymomonas mobilis* and type II L-asparaginase (ans B) from *Escherichia coli*

10 20 30 40 50 60

| | | | | |

ansB_E_coli MEFFKKTALAA--------LVMGFS---------GAALALPNITILATGGTIAGGGDSAT

tipoII_Z_mobilis_ZM4 MMIFKIPVKASSAAALAICMMMGATPAISMNNQVHSIQTLPRILVLATGGTISGKKNGMS

* :** .. *: ::** : : :**.* :*******:* :. :

Prim.cons. M22FK2222A2SAAALAIC22MG22PAISMNNQV22222LP2I22LATGGTI2G222222

70 80 90 100 110 120

| | | | | |

ansB_E_coli KSNYTVGKVGVENLVNAVPQLKDIANVKGEQVVNIGSQDMNDNVWLTLAKKIN--TDCDK

tipoII_Z_mobilis_ZM4 EIGYNAGGVTGKQLVEDIPELAKLAEINVEQIANIGSQDMNDAIWLRLAKRIQDAVAHNE

: .*..* * ::**: :*:* .:*::: **:.********* :** ***:*: . ::

Prim.cons. 222Y22G2V2222LV222P2L222A2222EQ22NIGSQDMND22WL2LAK2I2DA22222

130 140 150 160 170 180

| | | | | |

ansB_E_coli TDGFVITHGTDTMEETAYFLDLTVKCDKPVVMVGAMRPSTSMSADGPFNLYNAVVTAADK

tipoII_Z_mobilis_ZM4 ADGIVITHGTDTMEETAFFLDTVIRTDKPIILTGAMRPSTAIGADGPANLYEAIEVAATP

:**:*************:*** .:: ***:::.*******::.**** ***:*: .**

Prim.cons. 2DG2VITHGTDTMEETA2FLD22222DKP2222GAMRPST222ADGP2NLY2A222AA22

190 200 210 220 230 240

| | | | | |

ansB_E_coli ASANRGVLVVMNDTVLDGRDVTKTNTTDVATFKSVNYGPLGYIHNGKIDYQRTPARKHTS

tipoII_Z_mobilis_ZM4 KAKDHGVMIVMNDTIHAARWASKTHTTAVETFQSINAGPIGYVDPASVRFIEP---KKQP

: ::**::*****: .* .:**:** * **:*:* **:**:. ..: : .. *: .

Prim.cons. 22222GV22VMNDT2222R222KT2TT2V2TF2S2N2GP2GY22222222222PARK222

250 260 270 280 290 300

| | | | | |

ansB_E_coli DTPFDVSKLNELPKVGIVYNYANASDLPAKALVDAGYDGIVSAGVGNGNLYKSVFDTLAT

tipoII_Z_mobilis_ZM4 VPSYGLPTTAPLPAVEILYAHSGMGASIINDLIKTGVKGIILAGVGDGNSSKEAMAALNL

..:.:.. ** * *:* ::. . : *:.:* .**: ****:** *..: :*

Prim.cons. 22222222222LP2V2I2Y222222222222L222G22GI22AGVG2GN22K22222L22

310 320 330 340 350 360

| | | | | |

ansB_E_coli AAKTGTAVVRSSRVPTGATTQDAEVDDAKYGFVASGTLNPQKARVLLQLALTQTKD-PQQ

tipoII_Z_mobilis_ZM4 AVKQGVIVVRSSRTGSGFVNRNVEVNDDKNDFVVSYDLSPQKARILLQILIANGKNKLSD

*.* *. ******. :* ..::.**:* * .**.* *.*****:***: ::: *: .:

Prim.cons. A2K2G22VVRSSR222G222222EV2D2K22FV2S22L2PQKAR2LLQ222222K2K222

ansB_E_coli IQQIFNQY-

tipoII_Z_mobilis_ZM4 IQSAFEAGF

**. *:

Prim.cons. IQ22F222F

Global alignment of amino acid sequences of type II L-asparaginase (ans B) from *Zymomonas mobilis* and type II L-asparaginase (ans B) from *Erwinia chrysanthemi*

10 20 30 40 50 60

| | | | | |

tipoII_Z_mobilis_ZM4 MMIFKIPVKASSAAALAICMMMGATPAISMNNQVHSIQTLPRILVLATGGTISG-KKNGM

AnsB_erwinia_chrysanthemi -----MERWFKSLFVLVLFFVFTAS----------AADKLPNIVILATGGTIAGSAATGT

: .* .*.: ::: *: : :.**.*::*******:* .*

Prim.cons. MMIFK222222S222L2222222A2PAISMNNQVH2222LP2I22LATGGTI2GS222G2

70 80 90 100 110 120

| | | | | |

tipoII_Z_mobilis_ZM4 SEIGYNAGGVTGKQLVEDIPELAKLAEINVEQIANIGSQDMNDAIWLRLAKRIQDAVAHN

AnsB_erwinia_chrysanthemi QTTGYKAGALGVDTLINAVPEVKKLANVKGEQFSNMASENMTGDVVLKLSQRVNELLARD

. **:**.: . *:: :**: ***::: **::*:.*::*.. : *:*::*::: :*::

Prim.cons. 222GY2AG222222L2222PE22KLA2222EQ22N22S22M22222L2L22R22222A22

130 140 150 160 170 180

| | | | | |

tipoII_Z_mobilis_ZM4 EADGIVITHGTDTMEETAFFLDTVIRTDKPIILTGAMRPSTAIGADGPANLYEAIEVAAT

AnsB_erwinia_chrysanthemi DVDGVVITHGTDTVEESAYFLHLTVKSDKPVVFVAAMRPATAISADGPMNLLEAVRVAGD

:.**:********:**:*:**. .:::***:::..****:***.**** ** **:.**.

Prim.cons. 22DG2VITHGTDT2EE2A2FL222222DKP22222AMRP2TAI2ADGP2NL2EA22VA22

190 200 210 220 230 240

| | | | | |

tipoII_Z_mobilis_ZM4 PKAKDHGVMIVMNDTIHAARWASKTHTTAVETFQSINAGPIGYVDPASVRFIEPKKQPVP

AnsB_erwinia_chrysanthemi KQSRGRGVMVVLNDRIGSARYITKTNASTLDTFKANEEGYLGVIIGNRIYYQNRIDKLHT

:::.:***:*:** * :**: :**::::::**:: : * :* : : : : .: .

Prim.cons. 222222GVM2V2ND2I22AR222KT222222TF22222G22G222222222222222222

250 260 270 280 290 300

| | | | | |

tipoII_Z_mobilis_ZM4 S---YGLPTTAPLPAVEILYAHSGMGASIINDLIKTGVKGIILAGVGDGNSSKEAMAALN

AnsB_erwinia_chrysanthemi TRSVFDVRGLTSLPKVDILYGYQDDPEYLYDAAIQHGVKGIVYAGMGAGSVSVRGIAGMR

: :.: :.** *:***.:.. : : *: *****: **:* *. * ..:*.:.

Prim.cons. 2RSV22222222LP2V2ILY2222222222222I22GVKGI22AG2G2G22S2222A222

310 320 330 340 350 360

| | | | | |

tipoII_Z_mobilis_ZM4 LAVKQGVIVVRSSRTGSGFVNRNVEVNDDKNDFVVSYDLSPQKARILLQILIANGKNKLS

AnsB_erwinia_chrysanthemi KAMEKGVVVIRSTRTGNGIVPPDEELPG-----LVSDSLNPAHARILLMLALTR-TSDPK

*:::**:*:**:***.*:* : *: . :** .*.* :***** : ::. ... .

Prim.cons. 2A222GV2V2RS2RTG2G2V2222E222DKNDF2VS22L2P22ARILL222222G22222

370

|

tipoII_Z_mobilis_ZM4 DIQSAFEAGF

AnsB_erwinia_chrysanthemi VIQEYFHTY-

**. *.:

Prim.cons. 2IQ22F222F
